# The severity of depressive symptoms as a mediator in the sleep-osteoarthritis risk pathway: insights from the ELSA cohort

**DOI:** 10.3389/fnut.2025.1676763

**Published:** 2025-10-13

**Authors:** Junbo Wang, Lincheng Duan, Xin Duan, Li Chen, Zhichao Chen

**Affiliations:** ^1^Department of Orthopedics, Chengdu Integrated TCM and Western Medicine Hospital, Chengdu, China; ^2^Chengdu University of Traditional Chinese Medicine, Chengdu, China

**Keywords:** sleep quality, sleep duration, osteoarthritis, ELSA, cohort study

## Abstract

**Background:**

Osteoarthritis (OA) is a major cause of disability in middle-aged and older adults, yet its risk factors and mechanisms require further investigation. The connection between sleep disturbances and OA risk remains controversial, with underlying mechanisms unclear. The research aimed to examine the prospective association between sleep quality, sleep duration, and incident OA, and to evaluate whether the severity of depressive symptoms partially mediate this association.

**Methods:**

This analysis included 4,147 ELSA participants aged ≥50 years without baseline OA. Sleep quality (good/intermediate/poor) and duration (short: <7 h, optimal: 7-8 h, long: >8 h) were assessed via questionnaires. The severity of depressive symptoms was measured using the CES-D scale. Incident OA was determined by self-reported physician diagnosis. Multivariable Cox regression modeled associations between sleep (quality/duration) and OA risk. Threshold analysis and restricted cubic splines (RCS) explored the dose–response relationship for sleep duration. Mediation analysis quantified the severity of depressive symptoms’ role in the sleep-OA connection.

**Results:**

During 102 months of follow-up, 1,333 new OA cases were reported. Cox regression showed that intermediate and poor sleep quality significantly increased OA risk (HR = 1.23 and 1.74, respectively). RCS analysis revealed a U-shaped curve, with the lowest OA risk at 8 h of sleep. Short sleep (<7 h) was associated with higher OA risk (HR = 1.21), while long sleep (>8 h) showed no significant association. The severity of depressive symptoms mediated the relationship between both sleep quality and sleep duration with OA risk (mediation proportions: 22.39 and 22.11%, respectively). Sensitivity analyses confirmed result robustness.

**Conclusion:**

Poor sleep quality and short sleep duration are independent risk factors for incident OA in middle-aged and older adults. The severity of depressive symptoms partially mediates this relationship. Maintaining optimal sleep duration (8 h), improving sleep quality, and addressing depressive symptoms may help reduce OA risk.

## 1 Introduction

Osteoarthritis (OA) currently affects over 607 million people globally, with 73% of cases occurring in individuals aged 55 and older. The prevalence of OA continues to rise, driven not only by population aging and the obesity epidemic, but also by multiple other factors highlighted in recent evidence, including sedentary lifestyles, joint injuries, metabolic syndrome, poor diet quality, increased life expectancy, and multimorbidity, imposing a substantial public health and socioeconomic burden (1–5). While non-modifiable factors such as genetics contribute to OA susceptibility, modifiable risk factors—including obesity, biomechanical loading, and sleep disturbances—are increasingly recognized as important upstream targets for prevention (6). However, current management primarily focuses on weight control, exercise interventions, analgesics, and end-stage joint replacement, often overlooking non-articular factors—particularly the high prevalence of sleep disturbances as well as depressive symptoms among older adults. These factors can compromise analgesic efficacy, reduce rehabilitation adherence, and accelerate functional decline (7, 8).

Existing epidemiological evidence, predominantly from Asian and North American cohorts, presents relevant but geographically limited findings. Large-scale Chinese studies (CHARLS, CLHLS) associate increased sleep duration with a reduced risk of incident arthritis (9, 10), while short sleep duration (sleep duration of < 5 h per day) as well as poor sleep quality have been related to 38 and 56% increased arthritis risk, respectively (11). US NHANES data further suggest a U-shaped connection between sleep duration and OA, with both short (sleep duration of <7 h per day) and long sleep (sleep duration of ≥9 h per day) associated with approximately 18 and 19% elevated risk (12). Concurrently, depressive symptoms exhibit high comorbidity (20–30%) with both sleep disturbances and OA (13–15). Preclinical and clinical data indicate that depression activates the neuro-endocrine-immune axis (notably HPA-axis/sympathetic activation) and is accompanied by higher circulating IL-6, TNF-*α*, and CRP (16, 17). Together with the pro-inflammatory effect of sleep disturbance on IL-6/CRP (18), this pathway may mediate the detrimental impact of sleep abnormalities on musculoskeletal tissues (19); in OA, IL-6/TNF-*α* relate to pain and structural progression (20, 21), and IL-6 signaling promotes cartilage catabolism via MMP-13 and ADAMTS-5 (22, 23).

Recent work includes formal mediation analyses primarily addressing the sleep–depression–pain pathway. For example, in an OA cohort, depressive symptoms mediated the cross-sectional association between sleep disturbance and pain (24); in a longitudinal cohort, insomnia and short sleep predicted incident multisite musculoskeletal pain over 6 years with partial mediation by depressive symptoms (25); and serial pathways via sleep efficiency have been observed for knee OA pain (26). However, most prior studies are cross-sectional, focus on pain severity rather than incident OA, and rarely implement temporally ordered longitudinal mediation, thereby constraining causal inference and generalizability.

To address these gaps, this study leverages five waves of nationally representative longitudinal data from the ELSA. We aim to systematically assess the prospective impact of sleep quality as well as duration on the risk of incident physician-diagnosed OA in middle-aged and older adults. Furthermore, we will employ mediation analysis to investigate the potential mediating role of the severity of depressive symptoms in this connection. Elucidating this modifiable sleep-depression-OA pathway could inform early screening, comprehensive lifestyle interventions, and integrated psycho-orthopaedic preventive strategies, facilitating a shift from a purely “joint-centric” approach toward a holistic model of aging health.

## 2 Methods

### 2.1 Study design and population

**Figure 1:**
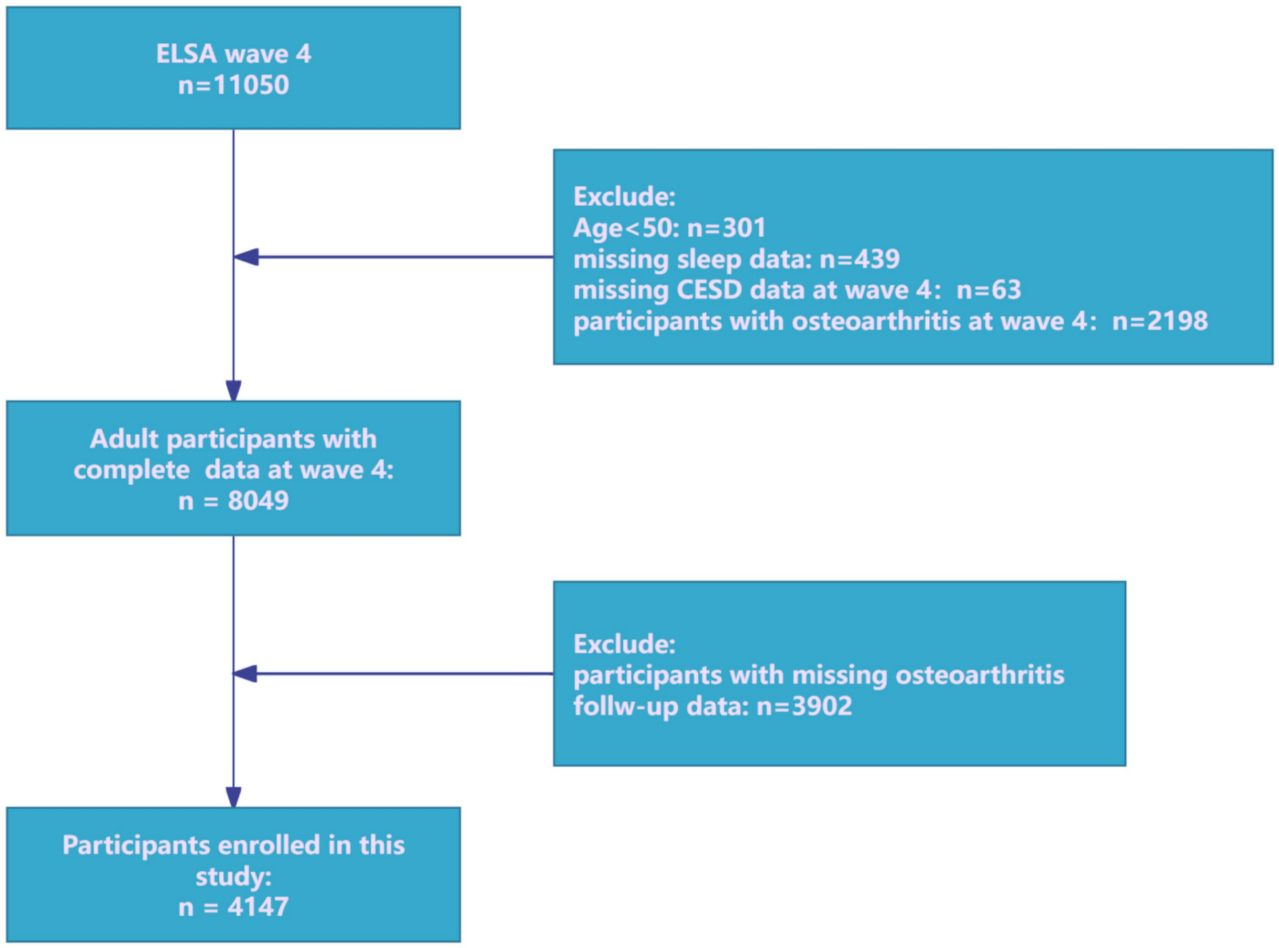
Research flowchart.

The prospective cohort research utilized data from the ELSA, a nationally representative panel research tracking multidimensional aspects of health, socioeconomic characteristics, and lifestyles in community-dwelling adults aged 50 years and older (27). In ELSA, participants receive an advance letter, are interviewed in their homes using computer-assisted personal interviewing (CAPI) approximately every two years, and complete a self-completion questionnaire. Wave 4 (2008) was designated as the baseline because it was the first time sleep-related data were assessed. In this analysis, individuals free of OA at Wave 4 (2008/09) were re-contacted and assessed at each biennial wave through Wave 9 (2018/19). Participants free of OA at baseline were rigorously selected and systematically followed through Wave 9. Although ELSA is designed to represent adults aged ≥50 years, cohabiting partners are interviewed irrespective of age. Consequently, a small number of participants aged <50 is present in the Wave-4 dataset. For this study, we pre-specified eligibility as age ≥50 at Wave 4 (2008) to align with the target population and survey weighting, and to ensure comparability of OA risk profiles. Participants aged <50 at Wave 4 were excluded. After applying inclusion as well as exclusion criteria (Figure 1), the final analytical cohort comprised 4,147 community-dwelling adults aged ≥50 years.

### 2.2 Assessment of exposures

*Sleep quality*: sleep quality was evaluated via three items from the modified Jenkins Sleep Questionnaire (difficulty falling asleep, waking up multiple times during the night, waking up feeling tired and exhausted) as well as one global sleep quality question (28, 29). Responses ranged from “not at all in the past month” (score = 1) to “more than three times a week” (score = 4). Total scores (range: 4–16; higher scores indicating worse quality) were categorized into three levels: Poor (≥12), Intermediate (8–11), and Good (4–7).

*Sleep duration*: self–reported average daily sleep duration (hours) was assessed. Participants were split into three groups: Short sleep (<7 h), Optimal sleep (7–8 h), as well as Long sleep (>8 h) (12, 30–32).

### 2.3 Mediator

Depressive symptoms were measured via the 8–item CES–D Scale, a widely leveraged and reliable instrument (33). Each “yes” response scored 1 point; each “no” response scored 0. Total scores ranged from 0 to 8, with higher scores indicating greater symptom severity.

### 2.4 Outcome

The outcome was the self–reported diagnosis of new–onset OA during follow–up. This was determined by asking, “Do you currently have osteoarthritis?” in the current wave and “Have you been diagnosed with osteoarthritis?” in the subsequent wave.

### 2.5 Covariates

A range of potential confounders that could affect the relationship between sleep and OA were also collected, including participants’ age, sex (male/female), race (White/non–White), education level (below high school/high school/college and above), marital status (Married/partnered vs. Never married/Separated/divorced/Widowed), BMI, smoking status (current smoker/former smoker/non–smoker), alcohol frequency (≥1 time/week vs. <1 time/week), physical activity level (moderate or vigorous exercise at least once a week: high/low), and chronic comorbidities including hypertension, diabetes, coronary heart disease (CHD), hyperlipidemia, and high cholesterol (yes/no). To maintain the integrity of the data, missing data were imputed via multiple imputations. The missing data proportions are shown in Supplementary Table 1.

### 2.6 Statistical analysis

Descriptive statistics were used to summarize the data, with variables reported as means ± standard deviations (SD), medians (interquartile range), or frequencies as well as percentages, depending on the data type. Chi–square tests were leveraged to analyze categorical variables, and one–way ANOVA or the Kruskal–Wallis test was leveraged to evaluate continuous variables with non–normal distribution. Missing covariate data were imputed using the R package mice with a random forest–based multiple imputation (MI) approach. Five imputed datasets (m = 5) were generated with 20 iterations to ensure algorithmic convergence. The results from these datasets were subsequently combined using Rubin’s rules to account for both within- and between-imputation variability.

Multivariable Cox proportional hazards regression models were leveraged to analyze the connection between sleep quality/sleep duration (as core independent variables) and OA risk (as a binary dependent variable: occurrence/not occurrence), while adjusting for potential confounders. The hazard ratios (HR) and 95% CI were calculated to assess the strength of the connection between sleep and OA risk. Proportional hazard assumption was examined using Schoenfeld residual methods. The Bonferroni correction used to adjust for multiple comparisons, the significance level was set at 0.025 (0.05 divided by 2 comparisons). The model construction followed a progressive adjustment strategy:

*Model 1*: Only sleep quality or sleep duration (unadjusted model).

*Model 2*: Adjusted for demographic variables (age, sex, race, marital status, education level).

*Model 3*: Fully adjusted for multidimensional covariates, including age, sex, race, marital status, education level, BMI (continuous variable), smoking status, alcohol frequency, physical activity level (high/low), as well as chronic disease status including hypertension, diabetes, CHD, hyperlipidemia, and high cholesterol (yes/no).

To explore the heterogeneity of associations, stratified subgroup analyses were conducted based on key demographic characteristics and lifestyle factors: age (50–64 years vs. ≥65 years), sex (male/female), education level (low/medium/high), marital status (married/cohabiting vs. single/divorced/widowed), BMI (<30 kg/m^2^ vs. ≥30 kg/m^2^), smoking status (current smoker/former smoker/non–smoker), and alcohol frequency (≥1 time/week vs. <1 time/week). Kaplan–Meier curves were plotted to assess the cumulative incidence of OA, as well as the log–rank test was used to evaluate survival curve differences between sleep quality and sleep duration groups.

Causal mediation analysis was conducted using the “mediation” package in R, to evaluate the mediating role of depressive symptoms in the relationship between sleep quality score and sleep duration with OA. The analysis was adjusted for gender, age, education level, marital status, smoking, alcohol, physical activity, BMI, as well as major chronic diseases (hypertension, diabetes, cardiovascular diseases, hyperlipidemia). Mediation effects were considered significant if the total effect of sleep on OA (path c) was significant, the effect of sleep on depressive symptoms (path a) was significant, and the effect of depressive symptoms on OA (path b) was significant. The indirect effect (a × b) with a 95% Bootstrap confidence interval (1,000 resampling) not containing 0 was considered evidence of mediation. The proportion mediated (PM) of the total effect was reported. All statistical analyses were conducted via R 4.4.3 software with a two-sided significance level of *α* = 0.05.

## 3 Results

### 3.1 Baseline characteristics

A total of 4,147 participants were included in this study, with a mean age of 62.62 (±7.74) years. Of these, 46.88% were male. Regarding sleep quality, 1,688 participants (40.70%) had good sleep quality, 1,743 (42.03%) had moderate sleep quality, and 716 (17.27%) had poor sleep quality (Table 1).

**Table 1:** Baseline characteristics of study populations across sleep quality.

Variable	Levels	*N*	Overall	Good	Intermediate	Poor	*p*-value
			*N* = 4,147	*N* = 1,688	*N* = 1,743	*N* = 716	
Age, mean (sd)		4,147	62.62 (7.74)	62.51 (7.75)	63.09 (7.78)	61.72 (7.55)	<0.001
BMI, mean (sd)		4,147	28.04 (4.92)	27.79 (4.75)	28.08 (4.87)	28.53 (5.40)	0.024
CESD, mean (sd)		4,147	1.06 (1.65)	0.46 (1.04)	1.01 (1.49)	2.60 (2.15)	<0.001
Follow–up duration (months), mean (sd)		4,147	102.11 (33.64)	105.90 (31.06)	101.52 (34.06)	94.61 (36.97)	<0.001
Gender, *n* (*p* %)		4,147					<0.001
	Female		2,203.00 (53.12%)	763.00 (45.20%)	956.00 (54.85%)	484.00 (67.60%)	
	Male		1,944.00 (46.88%)	925.00 (54.80%)	787.00 (45.15%)	232.00 (32.40%)	
Race, *n* (*p* %)		4,147					0.005
	Non–white		124.00 (2.99%)	65.00 (3.85%)	35.00 (2.01%)	24.00 (3.35%)	
	White		4,023.00 (97.01%)	1,623.00 (96.15%)	1,708.00 (97.99%)	692.00 (96.65%)	
Marital, *n* (*p* %)		4,147					<0.001
	Married/partnered		3,174.00 (76.54%)	1,342.00 (79.50%)	1,340.00 (76.88%)	492.00 (68.72%)	
	Never married/Separated/divorced/Widowed		973.00 (23.46%)	346.00 (20.50%)	403.00 (23.12%)	224.00 (31.28%)	
Education, *n* (*p* %)		4,147					<0.001
	Below high school		1,207.00 (29.11%)	457.00 (27.07%)	491.00 (28.17%)	259.00 (36.17%)	
	College or above		1,960.00 (47.26%)	858.00 (50.83%)	827.00 (47.45%)	275.00 (38.41%)	
	High school		980.00 (23.63%)	373.00 (22.10%)	425.00 (24.38%)	182.00 (25.42%)	
Smoke, *n* (*p* %)		4,147					0.009
	Former		433.00 (10.44%)	180.00 (10.66%)	178.00 (10.21%)	75.00 (10.47%)	
	No		3,207.00 (77.33%)	1,301.00 (77.07%)	1,379.00 (79.12%)	527.00 (73.60%)	
	Yes		507.00 (12.23%)	207.00 (12.26%)	186.00 (10.67%)	114.00 (15.92%)	
Drink, *n* (*p* %)		4,147					<0.001
	< Once a week		1,635.00 (39.43%)	586.00 (34.72%)	690.00 (39.59%)	359.00 (50.14%)	
	> = Once a week		2,512.00 (60.57%)	1,102.00 (65.28%)	1,053.00 (60.41%)	357.00 (49.86%)	
Diabetes, *n* (*p* %)		4,147					0.031
	No		3,786.00 (91.29%)	1,561.00 (92.48%)	1,586.00 (90.99%)	639.00 (89.25%)	
	Yes		361.00 (8.71%)	127.00 (7.52%)	157.00 (9.01%)	77.00 (10.75%)	
High cholesterol, *n* (*p* %)		4,147					<0.001
	No		3,029.00 (73.04%)	1,301.00 (77.07%)	1,246.00 (71.49%)	482.00 (67.32%)	
	Yes		1,118.00 (26.96%)	387.00 (22.93%)	497.00 (28.51%)	234.00 (32.68%)	
Cancer, *n* (*p* %)		4,147					0.020
	No		3,875.00 (93.44%)	1,599.00 (94.73%)	1,611.00 (92.43%)	665.00 (92.88%)	
	Yes		272.00 (6.56%)	89.00 (5.27%)	132.00 (7.57%)	51.00 (7.12%)	
CHD, *n* (*p* %)		4,147					0.002
	No		3,922.00 (94.57%)	1,621.00 (96.03%)	1,634.00 (93.75%)	667.00 (93.16%)	
	Yes		225.00 (5.43%)	67.00 (3.97%)	109.00 (6.25%)	49.00 (6.84%)	
Hypertension, *n* (*p* %)		4,147					0.138
	No		2,229.00 (53.75%)	937.00 (55.51%)	923.00 (52.95%)	369.00 (51.54%)	
	Yes		1,918.00 (46.25%)	751.00 (44.49%)	820.00 (47.05%)	347.00 (48.46%)	
OA, *n* (*p* %)		4,147					<0.001
	No		2,814.00 (67.86%)	1,252.00 (74.17%)	1,167.00 (66.95%)	395.00 (55.17%)	
	Yes		1,333.00 (32.14%)	436.00 (25.83%)	576.00 (33.05%)	321.00 (44.83%)	

### 3.2 Association between sleep quality/duration and OA

When sleep quality score was analyzed as a continuous variable, a significant negative correlation with OA risk was observed in unadjusted, partially adjusted, and fully adjusted models: Hazard ratios (HR) and 95% confidence intervals (CI) were 1.08 (1.06–1.10), 1.07 (1.05–1.09), and 1.06 (1.05–1.08), respectively (Table 2). When sleep quality was treated as a categorical variable, a consistent trend was observed: after full adjustment, the risk of OA in the moderate sleep quality group was 23% higher than in the good sleep quality group (HR = 1.23, 95% CI: 1.09–1.40), and the risk in the poor sleep quality group was 74% higher (HR = 1.74, 95% CI: 1.50–2.02), with a significant dose–response trend (*P* for trend < 0.001).

**Table 2:** Cox proportional hazards estimates for the association of sleep quality and sleep duration with incident OA.

Variables	Model 1			Model 2			Model 3	
	HR (95%CI)	*P*		HR (95%CI)	*P*		HR (95%CI)	*P*
Sleep quality scores	1.08 (1.06 ~ 1.10)	**<0.001**		1.07 (1.05 ~ 1.09)	**<0.001**		1.06 (1.05 ~ 1.08)	**<0.001**
Sleep quality								
Good	1.00 (Reference)			1.00 (Reference)			1.00 (Reference)	
Intermediate	1.34 (1.18 ~ 1.51)	**<0.001**		1.26 (1.11 ~ 1.43)	**<0.001**		1.23 (1.09 ~ 1.40)	**0.001**
Poor	1.98 (1.71 ~ 2.28)	**<0.001**		1.81 (1.56 ~ 2.10)	**<0.001**		1.74 (1.50 ~ 2.02)	**<0.001**
*P* for trend		**<0.001**			**<0.001**			**<0.001**
Sleep duration	0.88 (0.84 ~ 0.92)	**<0.001**		0.88 (0.85 ~ 0.92)	**<0.001**		0.89 (0.85 ~ 0.93)	**<0.001**
Ideal sleep (7–8 h)	1.00 (Reference)			1.00 (Reference)			1.00 (Reference)	
Short sleep (<7 h)	1.28 (1.15 ~ 1.43)	**<0.001**		1.25 (1.12 ~ 1.40)	**<0.001**		1.21 (1.08 ~ 1.36)	**0.001**
Long sleep (>8 h)	1.08 (0.86 ~ 1.37)	0.508		0.98 (0.77 ~ 1.24)	0.838		0.96 (0.76 ~ 1.22)	0.737

Good sleep quality = Reference, Ideal sleep = Reference. HR, Hazard Ratio; CI, Confidence Interval; OA, Osteoarthritis; CHD, Coronary Heart Disease; BMI, Body Mass Index. Model 1: Crude. Model 2: Adjust: age, sex, race, marital status, education level. Model 3: Adjust: age, sex, race, marital status, education level, BMI, smoking status, alcohol frequency, physical activity level, as well as chronic disease status including hypertension, diabetes, CHD, hyperlipidemia, and high cholesterol. The bolded values indicate that the *p*-values are less than 0.05.

**Figure 2:**
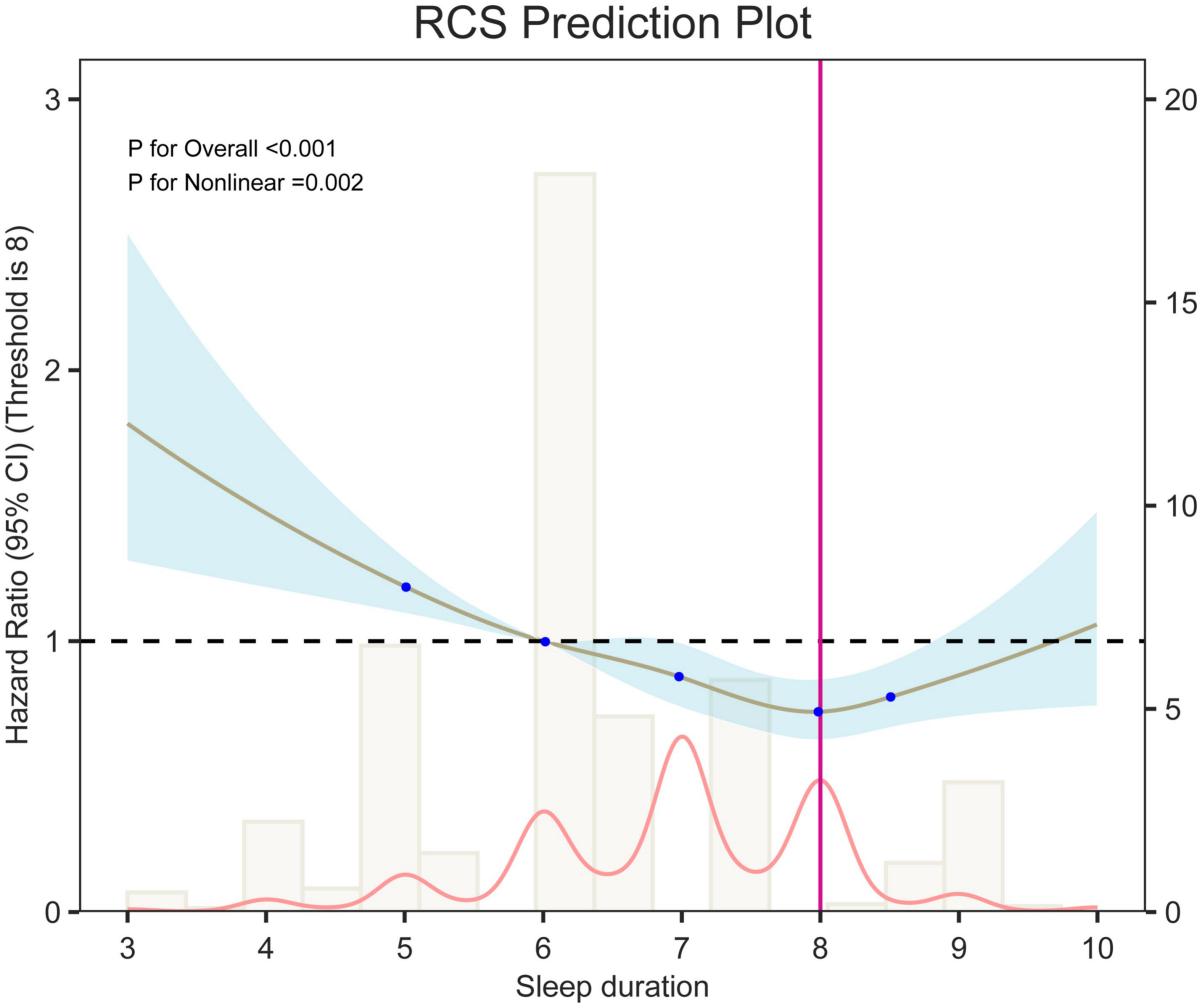
The RCS curve of the association between sleep duration and OA.

**Figure 3:**
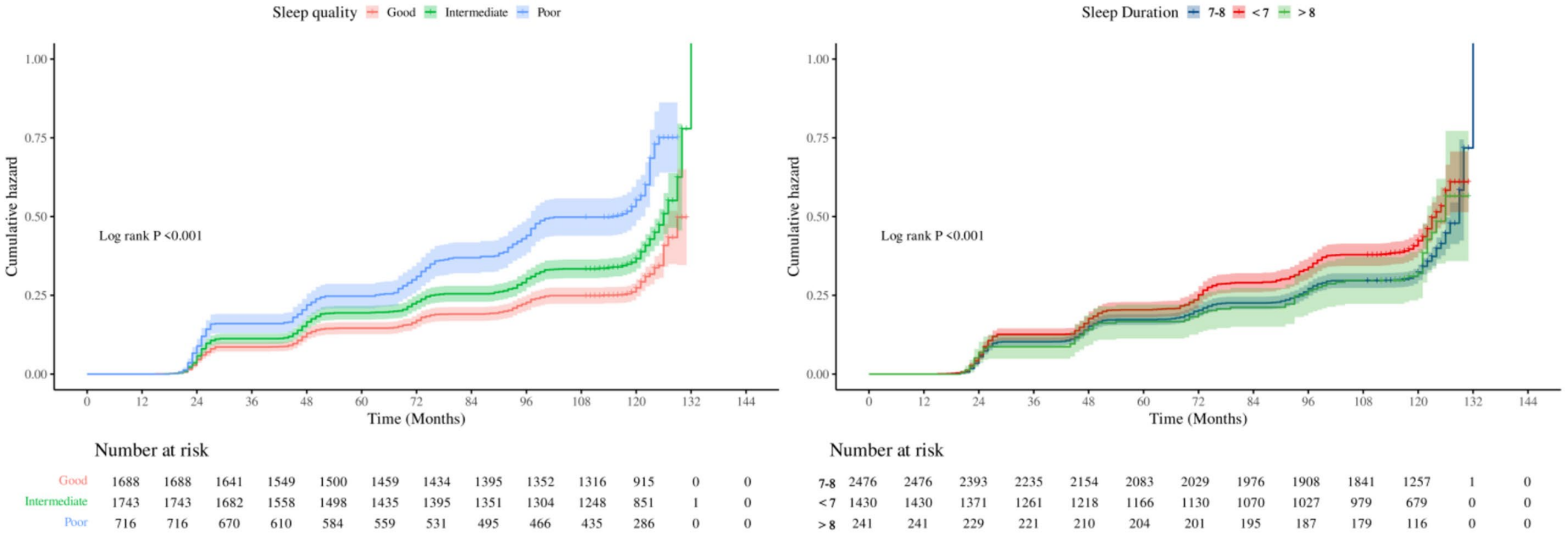
Kaplan–Meier curves for the cumulative incidence of OA.

Analysis of sleep duration showed that, overall, prolonged sleep was negatively related with OA risk (HR = 0.89, 95% CI: 0.85–0.93). In categorical comparisons, the short sleep group (<7 h) had a 21% higher risk (HR = 1.21, 95% CI: 1.08–1.36), while the long sleep group (>8 h) showed no significant change in risk. Restricted cubic spline (RCS) models (Figure 2) and threshold analysis (Supplementary Table 2) showed a non–linear connection between sleep duration and OA risk (overall *p* < 0.001, non–linear *p* < 0.01), with 8 h as the risk turning point: sleep duration of <8 h remarkably reduced the risk (HR = 0.85, 95% CI: 0.81–0.89), whereas >8 h increased the risk by 20% (HR = 1.20, 95% CI: 1.02–1.42). Cumulative incidence analysis for different sleep quality/duration groups showed trends similar to the main findings (Figure 3).

### 3.3 Mediating role of depressive symptoms

**Figure 4:**
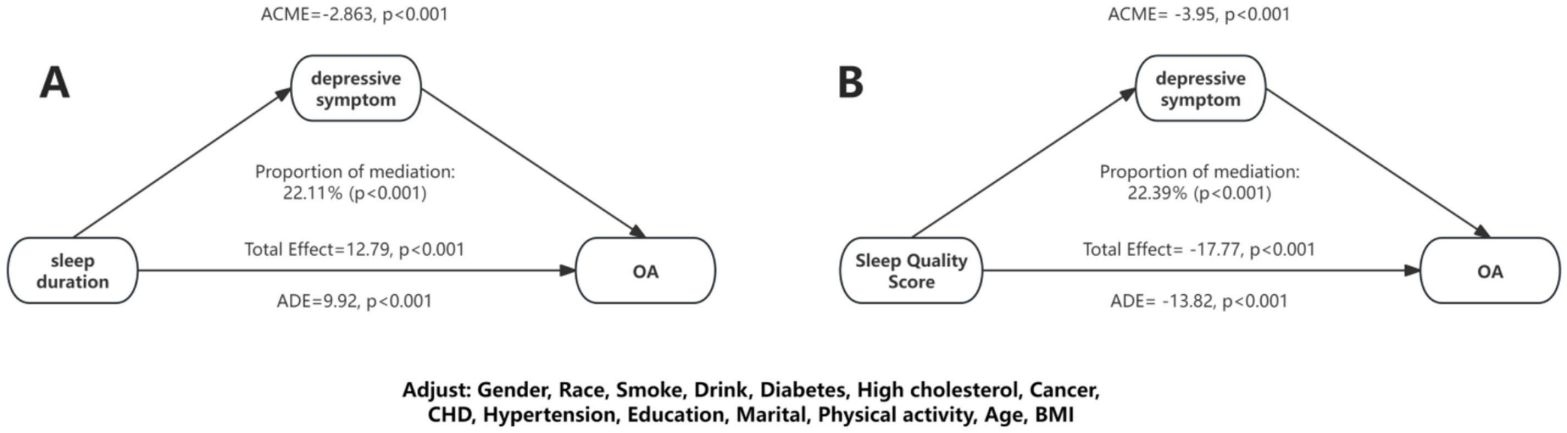
Mediation effects of depressive symptom on the association between sleep duration/sleep quality and OA.

Furthermore, a mediation analysis was conducted to explore the mediating effect of depressive symptoms. Figure 4 showed the mediating role of depressive symptoms in the relationship between sleep duration and OA. Depressive symptoms significantly mediated the association between sleep duration and OA, explaining 22.11% of the connection (*p* < 0.05). Additionally, we performed mediation analysis on the effect of depressive symptoms in the connection between sleep duration and OA. As shown in Figure 3, depressive symptoms explained 22.39% of the association.

### 3.4 Subgroup analyses

**Figure 5:**
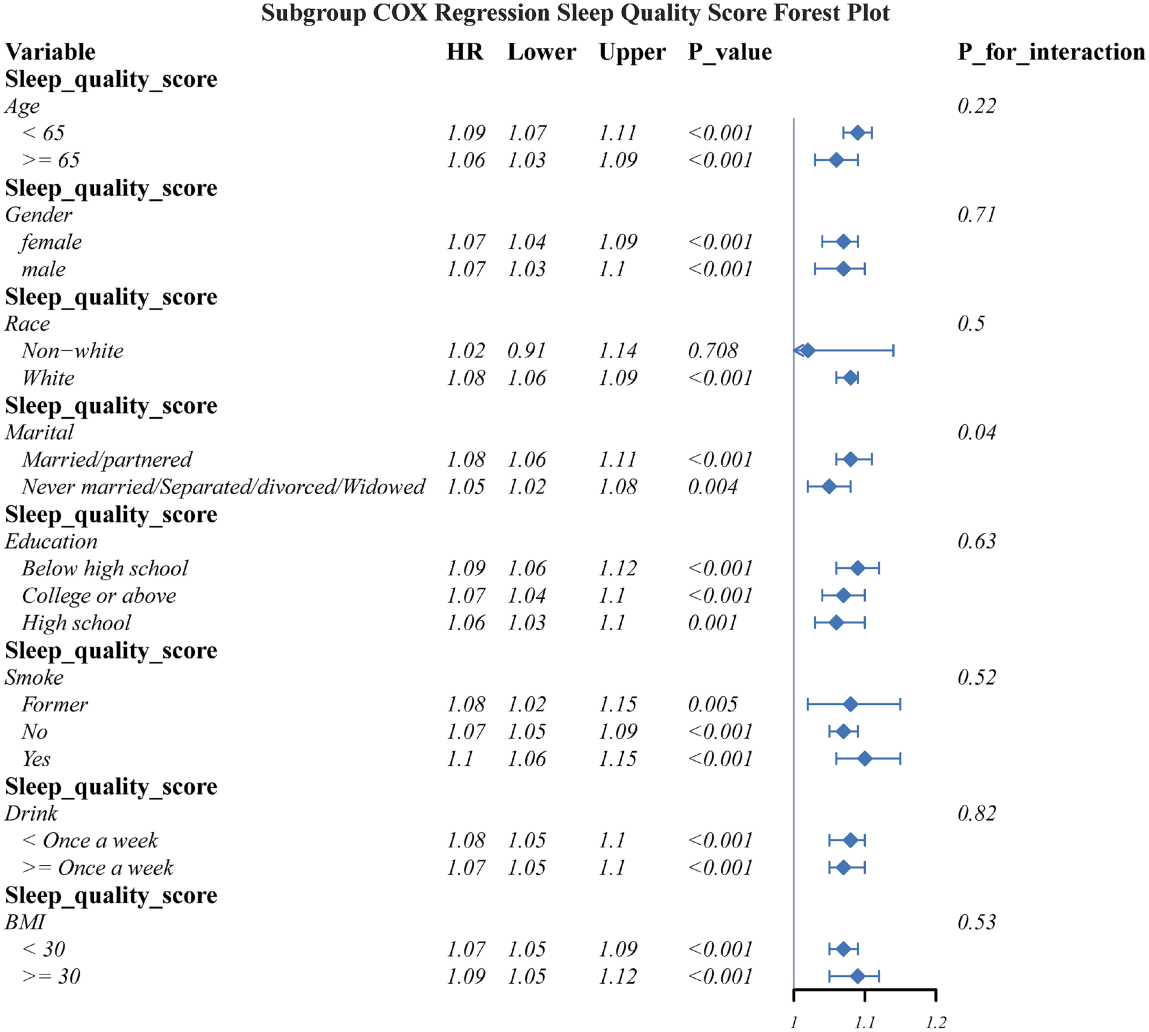
The association between sleep quality and OA in different subgroups.

**Figure 6:**
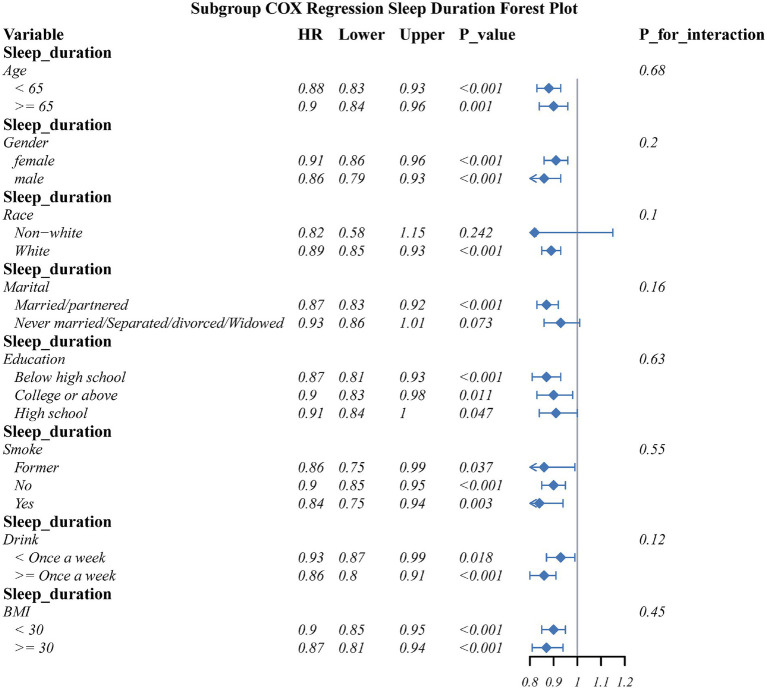
The association between sleep duration and OA in different subgroups.

Subgroup analyses assessed the connection of sleep quality score as well as sleep duration with OA risk across key demographic and lifestyle strata (Figures 5, 6). The detrimental effects of poor sleep quality as well as short sleep duration on OA risk were largely consistent across most subgroups. However, a significant interaction was observed for marital status in the connection between sleep quality and OA risk.

### 3.5 Sensitivity analyses

To verify the robustness of the results, two sensitivity analyses were performed: (1) we conducted a multivariable logistic regression analysis to assess the association between baseline sleep quality/duration and new–onset OA, adjusting for the same variables as in the Cox model (Supplementary Table 3); (2) to exclude reverse causality, OA cases from the first follow–up wave were removed, yielding similar results, which confirmed the robustness of the findings (Supplementary Table 4).

## 4 Discussion

The results of this study indicate that after adjusting for multiple confounders, compared to those with good sleep quality, individuals with moderate or poor sleep quality have a significantly increased risk of developing OA. There is a non–linear association between sleep duration and OA risk, exhibiting a “U–shaped” curve, with threshold analysis revealing that 8 h of sleep is associated with the lowest risk. Compared to the ideal sleep duration (7–8 h), shorter sleep (<7 h) is significantly associated with an increased risk of OA, while longer sleep durations show no significant association. Further mediation analysis indicates that depressive symptoms play a significant partial mediating role in the connection between sleep quality, sleep duration, and OA risk. Sensitivity analysis confirmed the robustness of the results. This suggests that maintaining an optimal sleep duration of 8 h, improving sleep quality, and addressing and managing depressive symptoms may help reduce the risk of OA in middle–aged and older adults.

This effect size aligns with the findings from the CHARLS cohort in China, which reported that short sleep (<6 h) increases the risk of knee OA by 72%, and the risk of OA is 88% higher among individuals with sleep disturbances occurring ≥5 days per week (10). It also corresponds with the CLHLS follow–up study, which showed that short sleep (<5 h) as well as poor sleep quality increase the incidence of OA by 56 and 38%, respectively (11). The NHANES data from the United States indicates a “U–shaped” connection between sleep duration and OA, with both short (<7 h) and long sleep associated with an 18–19% increased risk (12). Another 2024 Chinese prospective study found that each IQR increase in sleep duration could reduce the risk of new–onset OA by 16% (9). Additionally, Nordic primary care registry data show that 64–68% of knee/hip OA patients experience sleep problems, and 17–20% meet the criteria for insomnia (6), further highlighting the high comorbidity of sleep disorders. In terms of mental health, the CHARLS cohort presented a bidirectional relationship: knee OA predicts depression (HR = 1.38), and depression also predicts OA (HR = 1.51) (34). The OAI study found that mild depressive symptoms could accelerate the progression of early knee OA to joint replacement (35). Moreover, although Parmelee et al. did not conduct a formal mediation analysis, they observed—among 367 patients with physician-diagnosed knee osteoarthritis—that depressive symptoms appear to play an important role in the sleep–pain relationship, particularly at higher levels of pain severity (24).

Although our findings align with several large studies, effect sizes are not directly comparable. First, sleep measurement and thresholds are heterogeneous: cohorts use different cut-points for “short sleep” [e.g., <5 h (11), <6 h (10), <7 h (12)]. Although professional consensus recommends ≥7 h/night for adults (36), guideline targets do not necessarily coincide with analytic cut-points; moreover, sleep duration is typically self-reported, inviting non-differential measurement error that likely attenuates true effects. Second, outcome definitions differ: some studies analyse prevalence/progression or broad “arthritis,” rather than incident OA (11); in addition, knee and hip OA differ systematically in epidemiology and natural history/biology (biomechanics, susceptibility phenotypes, prognosis), precluding simple pooling (37). Third, study design and confounder control vary: several database analyses are cross-sectional, limiting temporal inference (12); even in prospective cohorts, objective sleep and repeated measures are often unavailable, and adjustment for physical activity, occupational/biomechanical load, adiposity, and comorbidity is inconsistent (11). Fourth, statistical implementation and reporting are non-uniform: ORs and HRs are mixed, adjustment sets differ, and tests for proportional hazards and competing risks are inconsistently reported—each affecting magnitude and interpretation (38, 39). Fifth, self-report misclassification likely biases toward the null: agreement between self-reported/clinical OA and radiographic OA is only moderate, and non-differential misclassification of binary variables typically attenuates associations (40, 41). Accordingly, while directional consistency across datasets is noteworthy, estimates should be interpreted cautiously within this heterogeneous methodological landscape.

Distinct from prior work centered on symptoms or function (e.g., sleep–pain links often interpreted via coefficient attenuation), our study addresses disease onset using time-to-event models and a counterfactual mediation approach, quantifying a partial mediation (22%) by depressive symptoms. This frames a modifiable pathway integrating sleep and mood into OA pathogenesis. Practically, the findings support concurrent management of sleep disturbance (e.g., CBT-I, circadian hygiene) and depressive symptoms (collaborative care, psychotherapy, activity prescription) in at-risk older adults to attenuate the indirect pathway—while recognizing that the 22% pertains to the HR scale and is not an absolute risk reduction, and that confirmatory pragmatic trials are needed.

Impaired sleep activates the hypothalamic–pituitary–adrenal axis (HPA) and sympathetic nerves, disrupts circadian rhythms, and is paralleled by upregulation of systemic inflammation (42–44). Multiple studies have shown that sleep disorders and/or abnormal sleep duration are associated with elevated interleukin-6 (IL-6) and C-reactive protein (CRP) (18), as well as consistently elevated IL-6/CRP in depressed populations (17, 18), suggesting that inflammatory response systems are activated and may be superimposed on abnormal sleep. Activated and may be superimposed on sleep abnormalities. Meanwhile, experimental and translational studies have demonstrated that IL-6 promotes MMP-13/ADAMTS-5 expression in chondrocytes (45) and reduces proteoglycan and type II collagen (21), and that systemic or localized blockade of IL-6/STAT3 attenuates cartilage destruction in models such as DMM (17, 46, 47); injection of IL-6 into the joint cavity induced cartilage degradation accompanied by upregulation of catabolic factors such as Mmp13 (22). In addition, sleep fragmentation and circadian dislocation are often accompanied by decreased nocturnal melatonin secretion (48–50); and melatonin can be induced by SIRT1-NF-κB (51), miR-146a/NRF2/HO-1 (52, 53), IRE1α-XBP1S-CHOP (54), PI3K/Akt and ERK (55) pathways exerted anti-inflammatory, antioxidant and rhythmic homeostasis maintenance, and protective effects on cartilage and subchondral bone, and the related interventions suppressed inflammation and matrix degradation in cellular and animal models (56). Thus, “HPA activation-cytokine upregulation-melatonin deficiency” constitutes a core biological pathway that points from sleep impairment to joint degeneration, and provides the basis for subsequent mechanisms (oxidative stress and reactive oxygen species) (57), epigenetic imbalances (58–60), gut-joint axis perturbations (61), subchondral *β*₂-adrenergic remodeling (62), and IDO-1/kynurenine metabolism imbalances (63, 64).

On top of that spine, depressive symptoms play a dual mediating role. In addition to the mediating role at the biological level mentioned above, depression is an important influencer at the behavioral level. Depression is associated with worsened sleep hygiene (prolonged sleep onset latency, daytime catch-up/rhythm disruption) (65–67), decreased physical activity (68, 69) with increased sedentariness (70), and nociception Catastrophising/negative mood hypersensitivity (71), amplifying the same-day and long-term effects of “poor sleep → high pain” (72, 73), and partially mediating depression in both OA and the general population (25, 74).

However, this study also has limitations: First, OA was self-reported. Relative to radiographic/clinical criteria, self-report generally shows high specificity but lower sensitivity, implying likely non-differential misclassification of incident OA that would bias associations toward the null. Second, measurement of sleep quality and depressive symptoms relied on questionnaires; lack of accelerometry/multichannel sleep monitoring, imaging-confirmed OA, and inflammatory/hormonal biomarkers limits mechanistic inference. Third, although we prespecified adjustment for major confounders, residual confounding (e.g., time-varying physical activity, early undiagnosed OA, analgesic use, care-seeking) cannot be excluded. Fourth, temporality: exposures and the mediator were assessed at baseline; we did not model time-varying trajectories, and protopathic effects are possible if subclinical OA affected sleep before clinical recognition. We mitigated this by excluding baseline OA and using incident events, but latency remains a consideration. Fifth, selection processes may affect generalizability: design-based exclusions (e.g., age <50), missing key measures, and wave-to-wave attrition from Wave 4 to the analytic cohort could introduce selection bias; multiple imputation addressed item missingness in covariates but does not remedy unit attrition. Population-level inferences should therefore be interpreted with caution. Future studies using survey weighting or inverse-probability-of-attrition weighting and, where feasible, linkage to administrative/registry data and repeated exposure/mediator measurements are warranted.

To address these limitations, future work should: (1) validate self-reported OA against radiographic/clinical standards and calibrate misclassification; (2) embed objective sleep (actigraphy/PSG), repeated measures of depression, and biomarkers (IL-6, CRP, cortisol, melatonin); (3) strengthen causal inference via prespecified DAGs, counterfactual mediation, and g-methods, with PH diagnostics and sensitivity analyses; (4) mitigate selection/temporality using survey/IPW weights, registry linkage, and lagged/landmark designs; and (5) test translation in pragmatic or factorial/SMART trials of combined sleep and depression management targeting the 22% mediated pathway, including cost-effectiveness and implementation outcomes.

## 5 Conclusion

In the ELSA cohort of middle–aged and older adults, poor sleep quality and insufficient sleep duration are independent risk factors for the onset of OA. Depressive symptoms partially mediate the association between sleep disturbances (both quality and duration) and increased OA risk. The findings suggest that maintaining an optimal sleep duration of 8 h, improving sleep quality, and addressing and managing depressive symptoms may help reduce OA incidence in middle–aged and older adults.

## Data Availability

The original contributions presented in the study are included in the article/Supplementary material, further inquiries can be directed to the corresponding author.
