# Supplementary material for: The severity of depressive symptoms as a mediator in the sleep-osteoarthritis risk pathway: insights from the ELSA cohort
Source: Front Nutr. 2025 Oct 13;12:1676763. doi: 10.3389/fnut.2025.1676763 (PMC12554558; doi:10.3389/fnut.2025.1676763)
Supplement: Supplementary file 1 [file Table_1.DOCX]

**Depressive Symptoms Mediate the Association Between Sleep Disturbances and Incident Osteoarthritis: Prospective Evidence from the ELSA Cohort**

**Supplemental Tables 1.** The numbers (percentages) of participants with missing covariates.

**Supplemental Tables 2.** Threshold effects of the association between sleep duration and OA.

**Supplemental Tables 3**. Logistic Regression Estimates of the Association between sleep quality, sleep duration and the Risk of incident OA.

**Supplemental Tables 4.** Association of sleep quality and sleep duration with risks of incident OA after excluding participants with incident OA during the first wave of follow-up.

**Supplemental Tables 1.** The numbers (percentages) of participants with missing covariates.

| **Covariate** | **Number** | **Percentage (%)** |
| --- | --- | --- |
| BMI | 237 | 12.95 |
| Current smoking | 24 | 0.58 |
| Alcohol consumption | 377 | 9.09 |
| Education | 315 | 7.6 |
| Race | 1 | 0.02 |

**Supplemental Tables 2.** Threshold effects of the association between sleep duration and OA.

| VariableName | RowName | Value | CI_2.5 | CI_97.5 | Pvalue |
| --- | --- | --- | --- | --- | --- |
| Sleep_duration | Model1 Line Effect | 0.8770 | 0.8386 | 0.9173 | 0.0000 |
|  | Model2 Threshold(W) | 8 |  |  |  |
|  | Model2 <W Effect | 0.8455 | 0.8056 | 0.8873 | 0.0000 |
|  | Model2 >W Effect | 1.2017 | 1.0176 | 1.4191 | 0.0304 |
|  | Log-likelihood ratio test | 0.0005 |  |  |  |

**Supplemental Tables 3**. Logistic Regression Estimates of the Association between sleep quality, sleep duration and the Risk of incident OA.

| Variables | Model1 | |  | Model2 | |  | Model3 | |
| --- | --- | --- | --- | --- | --- | --- | --- | --- |
|  | OR (95%CI) | *P* |  | OR (95%CI) | *P* |  | OR (95%CI) | *P* |
| Sleep quality scores | 1.10 (1.08 ~ 1.13) | **<.001** |  | 1.09 (1.07 ~ 1.11) | **<.001** |  | 1.08 (1.06 ~ 1.11) | **<.001** |
| Sleep quality |  |  |  |  |  |  |  |  |
| Good | 1.00 (Reference) |  |  | 1.00 (Reference) |  |  | 1.00 (Reference) |  |
| Intermediate | 1.42 (1.22 ~ 1.64) | **<.001** |  | 1.33 (1.15 ~ 1.55) | **<.001** |  | 1.30 (1.12 ~ 1.51) | **<.001** |
| Poor | 2.33 (1.94 ~ 2.80) | **<.001** |  | 2.11 (1.75 ~ 2.55) | **<.001** |  | 2.01 (1.66 ~ 2.44) | **<.001** |
| P for trend |  | **<.001** |  |  | **<.001** |  |  | **<.001** |
| Sleep duration | 0.85 (0.81 ~ 0.90) | **<.001** |  | 0.86 (0.81 ~ 0.91) | **<.001** |  | 0.86 (0.82 ~ 0.91) | **<.001** |
| Ideal sleep (7–8 h) | 1.00 (Reference) |  |  | 1.00 (Reference) |  |  | 1.00 (Reference) |  |
| Short sleep (<7 h) | 1.36 (1.19 ~ 1.57) | **<.001** |  | 1.33 (1.15 ~ 1.53) | **<.001** |  | 1.28 (1.11 ~ 1.48) | **<.001** |
| Long sleep (>8 h) | 1.11 (0.84 ~ 1.48) | 0.455 |  | 0.98 (0.73 ~ 1.31) | 0.897 |  | 0.97 (0.73 ~ 1.31) | 0.862 |
| OR: Odds Ratio, CI: Confidence Interval | | | | | | | | |
| Model1: Crude | | | | | | | | |
| Model2: Adjust: Gender, Race, Education, Marital, Age | | | | | | | | |
| Model3: Adjust: Gender, Race, Smoke, Drink, Diabetes, High_cholesterol, Cancer, CHD, Hypertension, Education, Marital, Physical_activity, Age, BMI | | | | | | | | |

**Supplemental Tables 4.** Association of sleep quality and sleep duration with risks of incident OA after excluding participants with incident OA during the first wave of follow-up.

| Variables | Model1 | |  | Model2 | |  | Model3 | |
| --- | --- | --- | --- | --- | --- | --- | --- | --- |
|  | HR (95%CI) | *P* |  | HR (95%CI) | *P* |  | HR (95%CI) | *P* |
| Sleep quality scores | 1.08 (1.06 ~ 1.11) | **<.001** |  | 1.07 (1.05 ~ 1.09) | **<.001** |  | 1.07 (1.04 ~ 1.09) | **<.001** |
| Sleep quality |  |  |  |  |  |  |  |  |
| Good | 1.00 (Reference) |  |  | 1.00 (Reference) |  |  | 1.00 (Reference) |  |
| Intermediate | 1.35 (1.16 ~ 1.57) | **<.001** |  | 1.28 (1.10 ~ 1.49) | **0.001** |  | 1.26 (1.08 ~ 1.47) | **0.003** |
| Poor | 2.03 (1.70 ~ 2.42) | **<.001** |  | 1.85 (1.55 ~ 2.22) | **<.001** |  | 1.80 (1.50 ~ 2.16) | **<.001** |
| P for trend |  | **<.001** |  |  | **<.001** |  |  | **<.001** |
| Sleep duration | 0.88 (0.83 ~ 0.93) | **<.001** |  | 0.88 (0.84 ~ 0.93) | **<.001** |  | 0.89 (0.84 ~ 0.94) | **<.001** |
| Ideal sleep (7–8 h) | 1.00 (Reference) |  |  | 1.00 (Reference) |  |  | 1.00 (Reference) |  |
| Short sleep (<7 h) | 1.31 (1.14 ~ 1.50) | **<.001** |  | 1.28 (1.12 ~ 1.47) | **<.001** |  | 1.25 (1.09 ~ 1.44) | **0.002** |
| Long sleep (>8 h) | 1.19 (0.91 ~ 1.57) | 0.211 |  | 1.08 (0.82 ~ 1.42) | 0.579 |  | 1.06 (0.80 ~ 1.40) | 0.671 |
| HR: Hazard Ratio, CI: Confidence Interval | | | | | | | | |
| Model1: Crude | | | | | | | | |
| Model2: Adjust: Gender, Race, Education, Marital, Age | | | | | | | | |
| Model3: Adjust: Gender, Race, Smoke, Drink, Diabetes, High_cholesterol, Cancer, CHD, Hypertension, Education, Marital, Physical_activity, Age, BMI | | | | | | | | |
